# Online exports and the skilled-unskilled wage gap

**DOI:** 10.1371/journal.pone.0232396

**Published:** 2020-05-22

**Authors:** Marcio Cruz, Emmanuel Milet, Marcelo Olarreaga

**Affiliations:** 1 World Bank, Washington, D.C., United States of America; 2 UFPR, Curitiba, Brazil; 3 University of Geneva, Geneva, Switzerland; 4 CEPR, Washington, D.C., United States of America; Universitat de Valencia, SPAIN

## Abstract

The development of the Internet is often seen as a source of demand for skilled workers and therefore a potential driver of the wage gap between skilled and unskilled workers. This paper focuses on the impact that international trade in online platforms has on the skilled-unskilled wage gap. Because online trade allows smaller firms with relatively more unskilled workers to access world markets, one can expect an expansion of online exports to reduce the wage gap. After correcting for potential endogeneity bias in a sample of 22 developing countries for which online trade and wage gap data can be matched, we find that a 1 percent increase in the share of online exports over GDP leads to a 0.01 percent decline in the skilled-unskilled wage gap.

## 1 Introduction

We explore the impact of online international trade on the wage gap between skilled and unskilled workers. In a sample of 22 developing countries for which we have data on cross border online trade, and labor surveys to estimate the wage gap, we find that a one percent increase in the share of online exports over GDP reduces the wage gap by 0.01 percent. This can be explained by the information providing mechanisms in online platforms that reduce the fixed cost of exporting, allowing smaller firms with a more unskilled workforce to export. We provide evidence supporting this mechanism as the reduction in the skilled-unskilled wage gap associated with online exports is larger in countries with a larger share of small firms.

**Fig 1:**
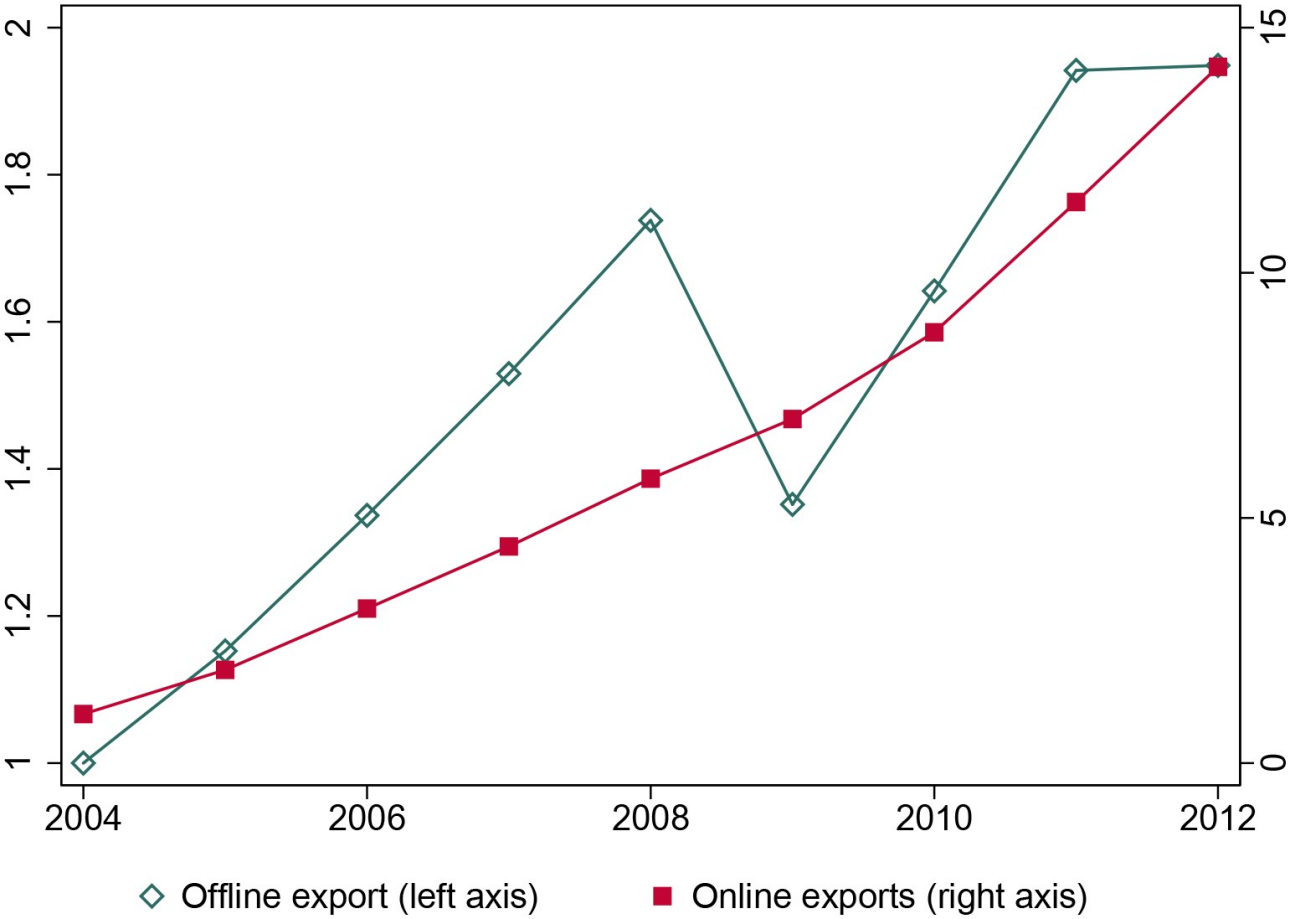
Evolution of world and online cross-border trade, 2004-2012. This is the partial plot of a regression of the log of the wage gap between skilled and unskilled workers on a time trend and country fixed effects for countries in our sample with more than 7 observations over the period 2004-2012.

**Fig 2:**
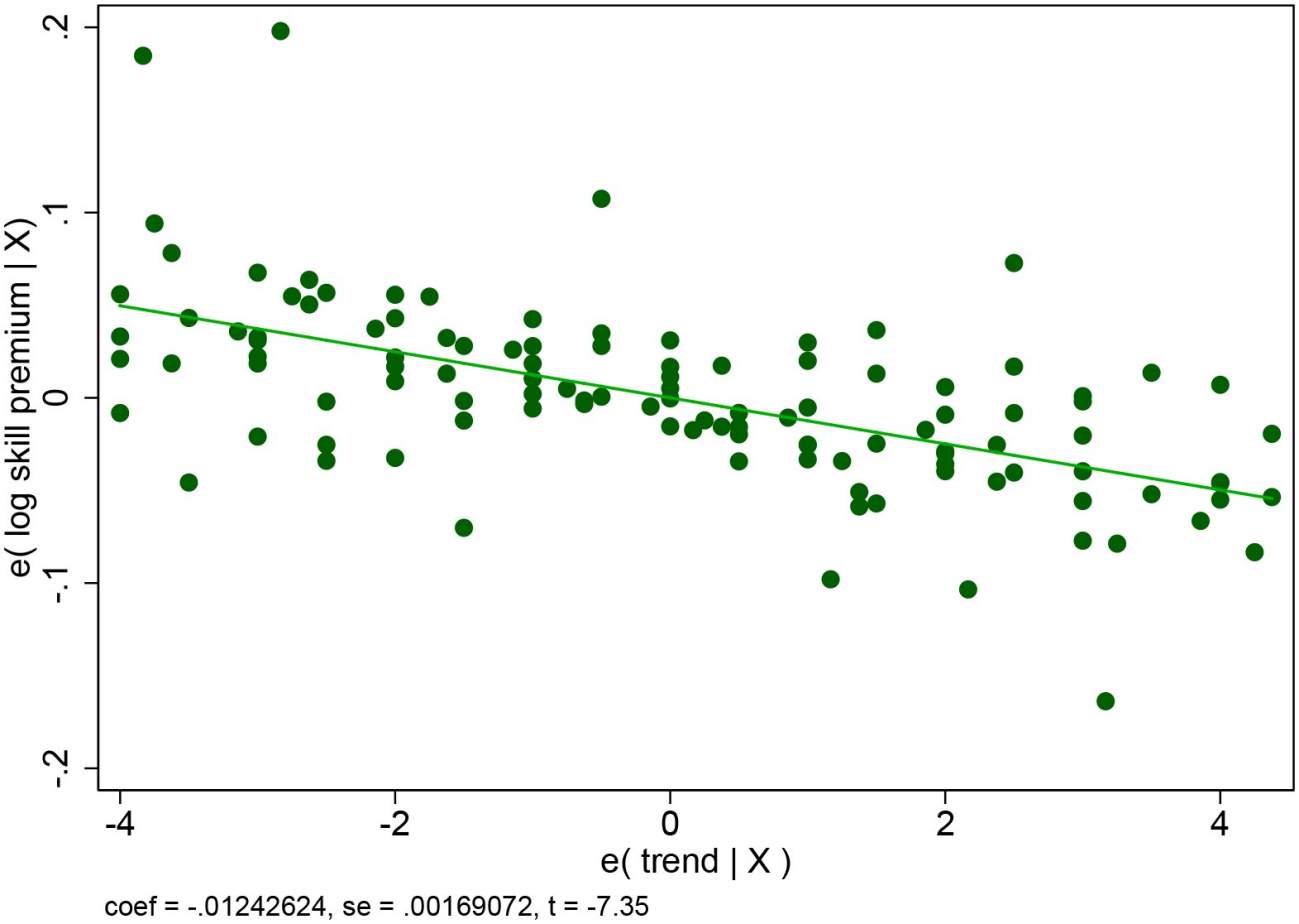
Evolution of wage skill premium, 2004-2012. This is the partial plot of a regression of the log of the wage gap between skilled and unskilled workers on a time trend and country fixed effects for countries in our sample with more than 7 observations over the period 2004-2012.

This result is important for at least three reasons. First, while world trade increased by 92 percent (8 percent annual growth) between 2004 and 2012, cross-border online trade grew more than 7 times faster (see Fig 1 where the left axis shows the growth in world trade, and the right axis the growth in online trade). Even if cross-border e-commerce only represents around 20 percent of online sales, it has been rapidly growing and its share is expected to double over the next 5 years [1]. For policy makers, it is important to assess early on the consequences of such a rapidly growing phenomenon. Second, the skilled-unskilled wage gap has been declining in the developing world over the last decade [2]. Our sample is no exception, with an average annual decline of 1 percent as can be seen from Fig 2. Understanding the forces behind the decline in the skilled-unskilled wage gap, and the role played by employment in small firms, can provide useful tools in the fight against income inequality. Third, it contributes to the debate on how technological progress affects skilled and unskilled workers, and ultimately income inequality. While the development of the internet and international trade has often been seen as skilled biased and contributing to income inequality (see for example, [3] for the internet and [4] or [5] for trade), this paper suggests that the combination of trade and the internet may have a different impact. Online platforms that allow small firms with a more unskilled intensive labor force to access world markets help unskilled workers and contribute to a reduction in income inequality. As noted by [6]: “*big enterprises were the first to reap the benefits of this technological progress. But the impact of information technology on small and medium-size enterprises may yet turn out to have the most impact on the economy*”. He also remarked more recently that small businesses are taking advantage of technologies which used to be the sole privilege of large companies: “*[…] even the smallest company can now afford a communications and computational infrastructure that would have been the envy of a large corporation 15 years ago*” [7].

In 2013 eBay integrated the service of an export intermediary to help firms reach consumers in foreign markets (the global shipping program). The program was intended to reduce the export administrative and logistic costs faced by exporting firms. [8] finds that the introduction of this program has significantly increase the probability of exporting of small and medium firms, but not of larger firms. Hui defines small firms as having total annual sales below 10,000USD. He finds that 71% of the increase in exports comes from small firms. He then provides strong evidence that this program has significantly reduced the export fixed entry cost.

We face several challenges. First, to date there is no official data on online international trade flows that is collected by any international agency, although some proposals have been put forward [1]. The main problem is finding a common definition of an online trade flow that could be used across all countries. For example should we consider ordering online, but paying offline an online transaction? There are no wrong or right answers to these questions, but a common framework is needed and is still to be determined. In this paper, we use as a proxy cross-border flows on the eBay platform for the period 2004-2012 which were made available by [9]. One problem with using eBay flows as a proxy is that the platform originally started as a peer-to-peer online market where second-hand products were auctioned, even if it rapidly became a platform mainly used by professional sellers on fixed price transactions. Because we want our proxy to reflect the access of firms to foreign consumers via an online platform rather than the general expansion of e-commerce, we disregard transactions that were undertaken under the auction system. We are still left with measurement error due to the fact that cross border eBay flows are only a fraction of total online transactions between firms and foreign consumers. We will address measurement error using an instrumental variable strategy described below.

The second challenge we face is omitted variable bias. Indeed, other unobserved factors such as technological progress may simultaneously determine online trade and the skilled-unskilled wage gap. To correct for this (and for measurement error) we use an instrumental variable estimator that uses demand shocks in trade partners as an instrument. Using the gravity setup that explains bilateral trade flows we use shocks in the importing country (i.e, importer-year fixed effects) to build an instrument for aggregate online exports in the exporting country. Other things equal, positive shocks in the *importing* country are expected to translate into greater exports from *exporting* countries. In addition, these shocks in *importing* countries are unlikely to be correlated with other determinants of the skilled-unskilled wage gap in *exporting* countries, although a full discussion of instrument validity is left for section 4.1.

The third challenge is to provide estimates of the wage gap between skilled and unskilled workers that are consistent across countries. We do this using data from the International Income Distribution Database from the World Bank. This database is a collection of labor and household surveys that have been harmonized to make cross-country analysis possible. We use this database to estimate consistent wage gaps between skilled and unskilled workers across countries and over time.

We are not the first to look at the determinants of the wage gap between skilled and unskilled workers. Labor economists have emphasized the role played by changes in supply and demand to explain changes in the wage gap and ultimately in wage inequality. The workhorse framework linking supply and demand to factor prices is developed in [10], later refined by several authors [see [11], for instance]. The most recent version of this model features skill-biased technologies, i.e. technologies that shift outward the relative demand for skilled workers. This skill-biased technology change (SBTC hereafter) induces a permanent shift in the demand for the labor service of skilled workers. The fact that wage inequalities increased at the same time when many industries were investing in computers gave strong support to the skill-biased technology change hypothesis [12–15]. However, technological change need not be biased in favor of skilled workers. [16] shows that as Europe was experiencing significant technological progress during the 500 years before the First World War, the wage gap between skilled and unskilled workers declined by more than 50%. In our empirical model we will control for both demand and supply side determinants of the wage gap. Our contribution is to add as potential demand driver the growth in online international trade.

The literature has also looked at the importance of trade as a determinant of the wage gap between skilled and unskilled workers. Trade economists have stressed the fact that the timing of rising wage inequalities in developed and developing countries coincided with a rapid increase in international trade flows [17, 18]. [19] find that imports of intermediate inputs account for about 15 percent of the increase in the relative wage of skilled workers in the United States over the period 1979-1990. SBTC is twice as important and accounts for about a third of the increase. Evidence from developing countries is more scarce, but also suggests that wage inequalities have been driven by episodes of trade liberalization in the 1980s and 1990s [20]. Lower tariffs imply that many developed economies outsourced part of their production process to lower income countries, which as emphasized by the “trading tasks” models [21, 22] can lead to increased wage inequalities in both developed and developing countries as long as the tasks being outsourced to developing countries are considered as unskilled in the rich country and skilled in the low-income country. [4] and [5] provide firm-level evidence for Argentina and Mexico, respectively, that exporting increases the skilled-unskilled wage gap within firms as it may require the adoption of new technologies, or quality upgrading, which increases the relative demand for skilled workers. [23] find that trade with China hurts more unskilled workers in the United States, and [24] and [25] argue that the negative impact of imports from China on wage and employment of unskilled workers in the United States is much larger than the impact of technological change.

More recently, income inequality has been declining in many developing countries, especially in Latin America [26]. [27] review some of this literature and conclude that the decline in overall inequalities in Latin American countries is to a large extent the consequence of a decline in the skilled-unskilled wage gap, income redistribution associated with government transfers, and the boom in commodity prices [28]. To sum up, the existing literature has concluded that both trade and skill biased technological progress play a role in explaining changes in the wage gap between skilled and unskilled workers. Importantly, these changes substantially contribute to the evolution in income inequality. However, to our knowledge the impact of online cross border trade on the wage gap between skilled and unskilled workers is yet to be investigated.

The rest of the paper is organized as follows. Section 2 presents the empirical strategy and section 3 the data. Section 4 discusses the empirical results of the impact of online exports on the wage gap. Section 5 explores theoretically and empirically the role played by small firms in explaining the impact of online exports on the wage gap. Section 6 concludes.

## 2 Empirical strategy

We explain variations in the wage skill gap within countries and over time with the following empirical model:

$$\begin{array}{ccc} \text{ln}{\left(wage\;gap\right)}_{c,t} & = & \delta_{c}+\eta_{t}+\beta_{1}\text{ln}{\left(Online\;exports/GDP\right)}_{c,t}+\beta_{2}\text{ln}{\left(Exports/GDP\right)}_{c,t}\\ & & +\beta_{3}\text{ln}{\left(Skilled/Unskilled\right)}_{c,t}\\ & & +\beta_{4}\text{ln}{\left(Internet\;users/Pop\right)}_{c,t}+\epsilon_{c,t} \end{array}$$ (1)

where *β*s are parameters to be estimated, *δ*_*c*_ and *η*_*t*_ are country and year fixed effects that control for time invariant country specific determinants of the wage gap such as labor institutions, and common determinants of the wage gap across countries such as technological change; *ϵ*_*c*,*t*_ is the error term. Total exports over GDP controls for the overall impact of trade openness on the skilled-unskilled wage gap. The expected sign is ambiguous as some of the early studies have shown that trade increased the wage gap, whereas some of the more recent studies have shown that the commodity boom has contributed to a reduction of the wage gap in developing countries. The relative supply of skilled workers (*Skilled/Unskilled*)_*c*,*t*_ should reduce the wage gap, and therefore *β*_3_ is expected to be negative. The share of Internet users in the population is a proxy for skilled biased technological change [3], and we therefore expect *β*_4_ to be positive.

Our coefficient of interest is *β*_1_, which captures the impact of online exports on the wage gap. We expect it to be negative. Indeed, the reduction in the fixed cost of entering foreign markets brought by online platforms helps entry into international markets by smaller and less productive firms with a relatively more unskilled-intensive labor force. For evidence that small firms have a more unskilled workforce, see the classic work by [29] or [30]. For evidence that online markets help small firms access otherwise unreachable international markets see [31]. They show that 85 percent of US firms on the eBay platform export and that their size is significantly smaller than the size of traditional US exporting firms. They also provide evidence suggesting that the larger export share is observed throughout the entire firm size distribution. [32] show that in developing countries the share of firms in online platforms that export tends to be larger than in the US. It is 100 percent in Chile, Indonesia, Jordan, Peru, Thailand, Ukraine and South Africa. Also, evidence from firm surveys by Ecommerce Europe suggest that more than 70 percent of firms selling online have less than 10 employees (see https://www.ecommerce-europe.eu/). Thus, as online markets develop, this provide small firms with better access to international markets, which in turn leads to an increase in the relative labor demand for unskilled workers. This should reduce the wage gap.

An important concern with the estimation of Eq (1) is the endogeneity of online and total exports flows. We address this by instrumenting online and total trade flows using shocks in importing countries as the exogenous source of variation. We instrument online and total exports using predicted flows obtained through the estimation of a gravity equation on importer-year and importer-exporter fixed effects. The idea is simple: foreign demand shocks are likely to affect online exports, but are hopefully uncorrelated with other determinants of the wage gap in the exporting country. For a full discussion of instrument validity see the robustness section.

More formally, we first estimate the following gravity equations for online and total exports:

$$\begin{array}{ccc} \text{ln}(online\;exports_{x,m,t}/GDP_{x,t}) & = & \gamma_{m,t}+\beta_{x,m}+\epsilon_{x,m,t} \end{array}$$ (2)

$$\begin{array}{ccc} \text{ln}(exports_{x,m,t}/GDP_{x,t}) & = & \zeta_{m,t}+\alpha_{x,m}+\mu_{x,m,t} \end{array}$$ (3)

where *online exports*_*x*,*m*,*t*_ and *exports*_*x*,*m*,*t*_ are respectively the online and total exports from country *x* to country *m* in year *t*. *β*_*x*,*m*_ and *α*_*x*,*m*_ control for time-invariant country-pair specific determinants of trade flows such as distance, colonial linkages, common language, etc. *γ*_*m*,*t*_ and *ζ*_*m*,*t*_ are importer-year specific shocks that are the source of the exogeneous variation in online and total exports; *ϵ*_*x*,*m*,*t*_ and *μ*_*x*,*m*,*t*_ are error terms.

We then use the estimates in Eqs (2) and (3) to predict the log of bilateral online and total exports. To construct the instruments that will be used to identify the impact of online trade on the wage gap, we need to aggregate the bilateral exports flows at the exporter and year level. To do so, we first take the exponential of the predicted bilateral trade flows estimated by Eqs (2) and (3). We then aggregate these trade flows at the exporter and year level by summing the trade flows over all importers each year. After taking logs we use these two variables to instrument the log share of online and total exports in GDP when estimating Eq (1).

## 3 Data

The variable of interest is the wage gap between skilled and unskilled workers. The data source is the International Income Distribution Database (I2D2) put together by the World Bank which compiles more than 1,000 household and labor force surveys from 164 countries over the period 1960-2014 covering more than 120 million individuals. The data have been harmonized to the extent possible ensuring that conceptual variables have similar meaning, allowing for cross-country analysis (see [33] and [34] for more information about the database). For the purpose of this study, we selected a restricted sample of 22 developing countries for which sufficient data are available (see Table 1 for a list of countries and surveys). We kept surveys for which individual information on gender, age, education achievement, and labor income (wage) was available and consistent over the period 2004-2012 for which we have cross-border trade data. We kept the working age population, and dropped individuals below 15 and above 64 years of age. This restriction does not affect our results. Individuals outside the [15-64] years old interval account for on average 4.7% of the population, and 4.6% of the total wage bill. Finally, we kept workers who reported being paid employees rather than self-employed. Wages are reported in various units depending on the country being surveyed. We converted them into monthly full-time equivalent. We exclude the top and bottom 1% of the wage distribution as non-response and under-reporting are typically quite severe among high-income households, and the bottom end of the distribution is likely to suffer from measurement error, misreporting, and omissions to consider financial transfers within the household (such as remittances for instance). There are five categories of educational achievement: no education or incomplete primary, complete primary, incomplete secondary, complete secondary, and post-secondary.

**Table 1:** List of the countries and survey years.

Country	# Surveys	Years available
ARG	7	2005, 2006, 2007, 2008, 2009, 2010, 2012
BOL	4	2005, 2007, 2008, 2009
BRA	8	2004, 2005, 2006, 2007, 2008, 2009, 2011, 2012
CHL	3	2006, 2009, 2011
COL	7	2004, 2005, 2006, 2007, 2008, 2009, 2010
CRI	6	2004, 2005, 2006, 2007, 2008, 2009
DOM	9	2004, 2005, 2006, 2007, 2008, 2009, 2010, 2011, 2012
ECU	8	2004, 2005, 2006, 2007, 2008, 2009, 2010, 2012
ETH	6	2005, 2006, 2009, 2010, 2011, 2012
GTM	3	2004, 2006, 2011
HND	7	2004, 2005, 2006, 2007, 2009, 2010, 2011
KHM	3	2006, 2008, 2012
LKA	2	2004, 2008
MEX	3	2008, 2010, 2012
NIC	2	2005, 2009
PAN	9	2004, 2005, 2006, 2007, 2008, 2009, 2010, 2011, 2012
PER	9	2004, 2005, 2006, 2007, 2008, 2009, 2010, 2011, 2012
PHL	8	2004, 2005, 2006, 2007, 2008, 2009, 2010, 2011
PRY	8	2004, 2005, 2006, 2007, 2008, 2009, 2010, 2012
RUS	6	2004, 2005, 2006, 2007, 2008, 2009
SLV	4	2004, 2005, 2006, 2007
URY	9	2004, 2005, 2006, 2007, 2008, 2009, 2010, 2011, 2012

We define skilled workers as workers with at least a complete secondary education. In unreported descriptive statistics, we show that the real wages of workers with complete secondary and post-secondary education follow similar trend over time, and so do the wages of workers with below complete secondary education. This similarity in the trends suggest that there is a higher degree of homogeneity within the skilled and unskilled workers rather than between them. The group of unskilled workers is made of individuals without education (below primary), incomplete primary and complete primary education. Following [10] and [35], wages and labor supply are defined in terms of *efficiency units* by adjusting for the composition of the workforce. To do so, we predict wages based on observable worker characteristics. We follow [35] and define 50 demographic groups of individuals based on the five education categories (no education or incomplete primary education, complete primary education, incomplete secondary education, complete secondary education, and post-secondary education), five job experience groups (0-9 years, 10-19 years, 20-29 years, 30-39 years, and more than 40 years), and gender (50 = 5 education groups×5 experience groups×2 genders). We regress individual log wage on the five education dummies (*Educ*_*i*_), an experience quartic, the full interaction between the education dummies and the experience quartic, a dummy for whether the individual lives in an urban area, and an industry dummy (*Sector*_*i*_). There are ten broad industries in the I2D2: agriculture, mining, manufacturing, public utilities, construction, retail and wholesale, transport, finance, public administration, and others. More formally, we estimate the following equation:

$$\begin{array}{ccc} \ln w_{i} & = & \sum_{i=1}^{5}\alpha_{i}Educ_{i}+\beta Exper_{i}+\delta Exper_{i}^{2}+\sum_{i=1}^{5}\gamma_{i}Educ_{i}\times Exper_{i}\\ & & +\sum_{i=1}^{5}\zeta_{i}Educ_{i}\times Exper_{i}^{2}+Urban_{i}+Sector_{i}+\varepsilon_{i} \end{array}$$ (4)

We estimate Eq (4) for men and women separately, and for each country×year separately as well. We predict wages for each education level, experience level (at 5, 15, 25, 35, and 45 years of experience, corresponding to the average experience in each experience group defined above), sector, and for individuals living in urban areas (we set *Urban*_*i*_ equal to one when predicting wages). We obtain a predicted wage for each of the 50 demographic groups (25 for men and 25 for women). We then aggregate the predicted wages to obtain a single predicted wage for skilled and unskilled workers. The predicted wage for skilled worker is the weighted average of predicted wages for workers with at least a complete secondary education (*Educ*_*i*_ ≥ 4). The weights we used are defined as the average labor share of each of the 50 demographic group in each sector over time. For instance, if the average share over time of labor force of workers with exactly complete secondary education (so considered as skilled workers), 35 years of experience in the manufacturing sector is equal to 2% of the labor force, we use this 2% as weight when aggregating the predicted wages of skilled workers.

The online trade data are borrowed from [9], where we only consider fixed price transactions, as the mechanism we have in mind does not go through the auctioning of second-hand goods. It contains bilateral cross-border flows on the eBay platform over the period 2004-2012. Cross border flows represented on average 20 percent of the total value of transactions on the eBay platform during this period. Total trade data come from United Nations’ Comtrade. As shown in Fig 1 online trade grew much faster than overall trade over the period 2004-2012.

**Fig 3:**
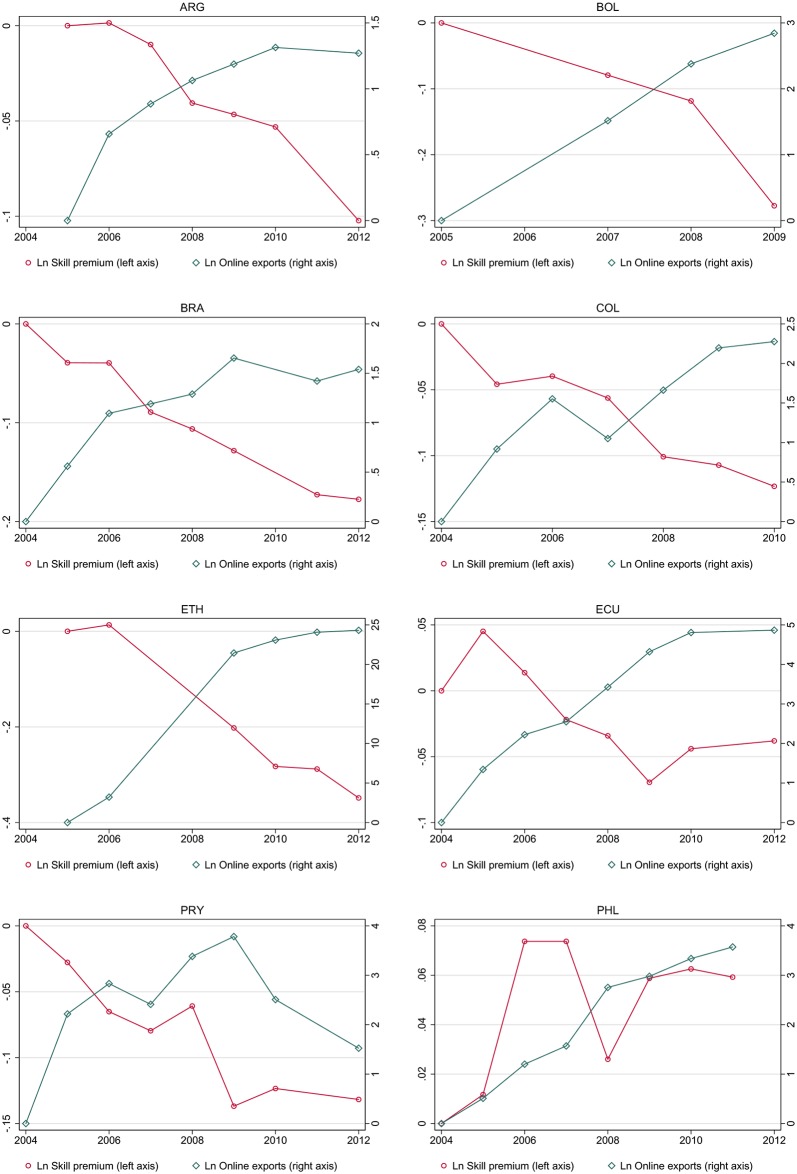
Wage gap in selected countries.

Fig 3 describes the evolution of the skilled-unskilled wage gap and online exports in some selected countries. Fig 3 suggests that as the wage gap between skilled and unskilled workers declined, online exports grew in most countries. But this is not the case in the Philippines, or at the end of the period in Paraguay for instance. Moreover, these are suggestive correlations that as discussed above do not necessarily imply any causal impact of online exports on the skilled-unskilled wage gap. Table 2 provides summary statistics for all the variables used in the estimations of Eqs (1)–(3). The data source for GDP and the share of Internet users is the World Bank’s World Development Indicators.

**Table 2:** Summary statistics.

Variable	#Obs.	Mean	Std. Dev.
Ln wage gap	131	0.46	0.20
Ln predicted online exports/GDP	131	-13.62	2.39
Ln online exports/GDP	131	-13.66	3.68
Ln predicted exports/GDP	131	-2.03	0.77
Ln exports/GDP	131	-1.83	0.69
Ln relative supply	131	-0.08	0.58
Internet user/Pop.	131	0.21	0.14

Source: Online trade data comes from [9]. Data on bilateral distance comes from [36]. Predicted trade flows are estimated by the authors. All other variables come from the World Bank’s World Development Indicators 2016.

## 4 Online exports and the wage gap

Table 3 presents the ordinary least square estimates. All regressions include country and year fixed effects, and standard errors are clustered at the country-level to account for intertemporal correlation in the error term for each country. In all columns online exports has a negative sign and is statistically significant at the 1 percent level. Using the estimate of column (5), which corresponds to Eq (1), we find that a 1 percent increase in the share of online exports over GDP decreases the wage gap by 0.01 percent. The log of total exports over GDP is never statistically significant. The coefficient on the relative supply of skilled workers is as expected negative and statistically significant. Its magnitude, -0.067 can be interpreted in terms of the elasticity of substitution between skilled and unskilled workers. Following a simple supply and demand framework *à la* [10], assuming that technological change is exogenous to the supply of skilled workers, the coefficient we estimate is (minus) the inverse of the elasticity of substitution between skilled and unskilled workers. Our estimate in column (4) therefore implies an elasticity around 15. The share of Internet users, which we use as a proxy for technological change, has a negative and statistically significant sign, suggesting that as the number of Internet users increases the wage gap between skill and unskilled workers declines.

**Table 3:** Impact of online exports on the wage gap (OLS).

	(1)	(2)	(3)	(4)	(5)
Ln online exports/GDP	-0.011***	-0.010***	-0.010***	-0.013***	-0.013***
	(0.002)	(0.001)	(0.001)	(0.002)	(0.002)
Ln total exports/GDP		-0.024*	-0.021*	-0.025	-0.022
		(0.013)	(0.011)	(0.016)	(0.015)
Ln relative supply			-0.062		-0.067**
			(0.041)		(0.030)
Internet user/Pop.				-0.250	-0.267
				(0.199)	(0.162)
Observations	131	131	131	131	131
*R*^2^	0.34	0.36	0.38	0.38	0.40
Country FE	Yes	Yes	Yes	Yes	Yes
Year	Yes	Yes	Yes	Yes	Yes

All regressions include country and year fixed effects. Standard errors in parenthesis clustered at the country level. Significance levels:

*: 10%,

**: 5%

***: 1%.

As discussed earlier an important problem with the estimates in Table 3 is the potential endogeneity of online exports due to measurement error as online exports are only captured by cross-border transactions that occurred over the eBay platform. Other platforms like Amazon, or Alibaba are excluded. Moreover, many electronic transactions do not occur over the platforms but directly on firms’ web pages, which do not have independent feedback mechanisms to create a reputation as a reliable business partner. There may also be omitted variable bias if the role of the Internet, which is imperfectly captured by the share of Internet users, is correlated with both online exports and the wage gap. In order to correct for these two problems we instrument online and total exports using Eqs (2) and (3).

After estimating Eqs (2) and (3) at the bilateral level, we predict export flows using importer-year and exporter-importer fixed effects. The correlation matrix of total and online export flows with their predicted values is given in Table 4. It is important to note that the correlation between the predicted export flows and the actual flows is always above 0.798, suggesting that our instrumental variables are doing a good job in explaining observed trade flows. It is also important to highlight that the correlations between the predicted online and total trade flows is positive, but not too high. It is 0.627 suggesting that the instruments are capturing different sources of variation in the determination of online and total trade flows.

**Table 4:** Correlation between observed and predicted trade flows.

	Ln X_*online*_/GDP	Ln $\hat{\text{X}{}_{online}}$/GDP	Ln X/GDP	Ln $\hat{\text{X}}$/GDP
Ln X_*online*_/GDP	1			
Ln $\hat{\text{X}{}_{online}}$/GDP	0.854	1		
Ln X/GDP	0.512	0.496	1	
Ln $\hat{\text{X}}$/GDP	0.661	0.627	0.798	1

X stands for total exports, and X_*online*_ for online exports. $\hat{\text{X}{}_{online}}$ and $\hat{\text{X}}$ are predicted online and total trade flows respectively.

First stage regressions are reported in Table 5 and show strong correlations between online or total exports and their respective instruments, but not across online and total exports. The F statistics are above 10 in all regressions. The R^2^ are also quite high for online exports at around 70%, but relatively low for total exports at around 21%. In column (1) we report first stage results for online exports using only the predicted online exports as an instrument. In columns (2) and (4) we report first stage results without controlling for the log of relative supply of skilled and unskilled labor, and the share of internet users in total population as arguably these variables may also be endogenous and introduce a bad control problem. Columns (3) and (5) report results using all instruments.

**Table 5:** First stage regressions.

*Dep. var*:	Ln $\frac{X_{online}}{GDP}$	Ln $\frac{X_{online}}{GDP}$	Ln $\frac{X_{online}}{GDP}$	Ln $\frac{X}{GDP}$	Ln $\frac{X}{GDP}$
	(1)	(2)	(3)	(4)	(5)
Ln predicted online exports/GDP	2.805***	2.423***	2.198***	-0.045	-0.045
	(0.128)	(0.378)	(0.399)	(0.030)	(0.051)
Ln predicted exports/GDP		1.294	1.058	0.535***	0.520***
		(1.505)	(1.344)	(0.154)	(0.136)
Ln relative supply			-1.268		0.200
			(0.859)		(0.338)
Internet user/Pop.			-12.116*		-0.018
			(5.919)		(2.074)
Observations	131	131	131	131	131
*R*^2^	0.69	0.70	0.73	0.21	0.21
Country FE	Yes	Yes	Yes	Yes	Yes
Year FE	Yes	Yes	Yes	Yes	Yes

All regressions include country and year fixed effects. Standard errors in parenthesis clustered at the country level. Significance levels:

*: 10%,

**: 5%

***: 1%.

Table 6 provides the instrumental variable estimates of Eq (1). All regressions include country and year fixed effects. In the first column we report results using only predicted share of online exports on GDP as an instrument for share of online exports on GDP, without controlling for total exports, nor other covariates. In columns (2) and (3) we report results with share of online and total exports on GDP instrumented by predicted share of online and total exports on GDP. Yet, in column (2) we do not control for the relative supply of skilled and unskilled workers, as well as the share of internet users in the population. Arguably these variables can be endogenous and we do not have any convincing instruments to correct for this. Therefore, to circumvent a bad control problem we provide results without the two control variables in the second column. Column (3) includes all variables from Eq (1). Note that in the three columns the models are just identified. In the first column we use one instrument (predicted online exports) and one endogenous variable (online exports). In the second and third column we have two instruments (predicted exports) and two endogenous variables (online and total exports).

**Table 6:** Impact of online exports on the wage gap (instrumental variables).

	(1)	(2)	(3)
Ln online exports/GDP	-0.012***	-0.008***	-0.010***
	(0.001)	(0.002)	(0.003)
Ln total exports/GDP		-0.120**	-0.125**
		(0.051)	(0.051)
Ln relative supply			-0.033
			(0.043)
Internet user/Pop.			-0.301
			(0.335)
Observations	131	131	131
Shea Partial r^2^ (predicted online exports)	0.601	0.358	0.354
Shea Partial r^2^ (predicted exports)		0.074	0.072
Test of excluded instruments F, Ln online exports/GDP (P-value)	0.000	0.000	0.000
Test of excluded instruments F, exports/GDP (P-value)		0.004	0.002
Anderson’s test (P-value)	0.000	0.004	0.005
Cragg–Donald test (P-value)	0.000	0.003	0.004
Country FE	Yes	Yes	Yes
Year FE	Yes	Yes	Yes

Column (1) uses only predicted online trade as an instrument. Columns (2) and (3) use predicted online and total trade flows as instruments. All regressions are just-identified. Results for column (1) are robust, with similar coefficient and statistical significance, if used predicted online and total trade flows as instruments. Shea’s partial R^2^ and the F test suggest that the instruments are relevant. The CraggDonald and Anderson’s tests reject the null hypothesis of under-identification. Standard errors in parenthesis clustered at the country level. Significance levels:

*: 10%,

**: 5%

***: 1%.

The estimated coefficients on the share of online exports are negative as expected and statistically significant at the 1 percent level. The estimates in column 1 suggest that a 1 percent increase in the share of online exports on GDP leads to a 0.01 percent decline in the wage gap. The estimated coefficient on the share of total exports on GDP is also always negative and statistically significant with a point estimate around -0.12. The coefficient on the relative supply of skilled workers remains negative. The share of Internet users is negative, but statistically insignificant. At the bottom of the table we report the p-value of the Anderson LM for the first stage which are strongly below the admitted threshold of 5%.

### 4.1 Robustness

The validity of our instruments for online and total exports requires that shocks to online and total imports in trading partners are uncorrelated with the error term in the wage gap equation for exporting countries. This may be a strong assumption if for example shocks in the importing country affect not only total and online imports, but also migration and capital flows, which can then affect the wage gap in exporting countries. The impact through migration is controlled for by the skilled to unskilled labor ratio, but the impact through capital flows is not controlled for in our main specification and can be biasing our results. Similarly, technology shocks in importing countries can affect online and total exports to these markets, but can also be correlated with technology changes in exporting countries which can affect their wage gap. The year fixed effects controls for this as long as these are common shocks across all exporting countries in our sample, but this is arguably a strong assumption.

To try to ensure that these alternative channels are not driving our results we provide two sets of robustness checks controlling for capital investment in the exporting country and as country-specific trends. Results are reported in Table 7. The first two columns report ordinary least square estimates and the last two columns instrumental variable results. Columns (1) and (3) control for country-specific trends and columns (2) and (4) control for capital investment in the exporting countries. Results are broadly consistent with the ones previously reported in Tables 3 and 6, even though the standard errors in column (3) need to be interpreted with care as the estimated covariance matrix of moment conditions is not of full rank. Results are also robust if we exclude the share of total exports as an explanatory variable and keep only the share of online exports on GDP, instrumented only by the predicted share of online exports on GDP, and the other covariates.

**Table 7:** Robustness check: Adding country trends, controlling for investment.

	(1)	(2)	(3)	(4)
Ln online exports/GDP	-0.025***	-0.013***	-0.017*	-0.013***
	(0.002)	(0.002)	(0.009)	(0.002)
Ln total exports/GDP	-0.056*	-0.051*	-0.145**	-0.084**
	(0.027)	(0.026)	(0.074)	(0.037)
Ln relative supply	0.068	-0.039	0.088	-0.023
	(0.070)	(0.027)	(0.064)	(0.030)
Internet user/Pop.	0.287	-0.430*	0.457	-0.559**
	(0.341)	(0.247)		
Net investment in non-financial assets (% of GDP)		0.009**		0.009***
		(0.003)		(0.003)
Observations	131	100	131	100
r^2^	0.88	0.43		
P-value Anderson canon corr. LR statistic			0.000	0.000
Country FE	No	Yes	No	Yes
Country time trends	Yes	No	Yes	No
Year FE	Yes	Yes	Yes	Yes

Columns (1) and (3) include country-specific linear time trends and year fixed effects. Column (2) and (4) include country and year fixed effects. The first two columns are estimated using ordinary least squares and the last two columns are estimated using instrumental variables. Standard errors in parenthesis clustered at the country level. Significance levels:

*: 10%,

**: 5%

***: 1%.

## 5 The role of small firms

As discussed earlier a potential explanation for the negative impact of online exports on the wage gap is that the reduction in the fixed cost of exporting benefits small firms which tend to be less skill-intensive. While small firms are under-represented in the population of regular exporters, anecdotal evidence suggest that they account for a significant share of the firms engaged in cross-border e-commerce. The company “Ecommerce Europe” which describes itself as “the voice of the e-commerce sector in Europe” represents over 25,000 online shops across Europe. Its 2016 e-commerce report surveyed 585 European firms engaged in e-commerce and shows that 334 of the surveyed firms are what they call “pure-players” (i.e. not retailers or wholesalers, see https://www.ecommerce-europe.eu/. Most of these independent firms (72%) have fewer than 10 employees and 84% have fewer than 20 employees, and two-thirds engage in cross-border e-commerce. In developing countries, anecdotal evidence taken from [1] and [37] shows that when facing substantial challenges while engaging in e-commerce, many micro and small enterprises rely on existing online platforms. Such platforms propose a variety of service that micro and small firms would not be able to provide on their own such as advertising, product management, shipping, and most importantly payments via credit cards, PayPal, or bank accounts. Empirical evidence from [38] on firms from 21 emerging economies also shows that compare to traditional exporters, firms exporting through eBay sell to more destinations, suggesting a lower export fixed cost.

We first develop a simple analytical model to analyze the role of small firms in explaining the impact of online exports on the wage gap. The model suggests that the reduction in the cost of exporting offered by online platforms has a stronger impact in countries with a large share of employment in small firms. We then empirically test this prediction.

### 5.1 A conceptual framework

Building on [39] and [40] it can be shown that a reduction in the fixed cost of exporting faced by small firms leads to a reduction in the wage gap between skilled and unskilled workers, and that this reduction is larger in the presence of a relatively larger share of small firms.

On the demand side we have a representative consumer with CES preferences over two imperfectly substitutable composite goods *X*_*h*_ and *X*_*l*_ who maximizes her utility with respect to her budget constraint *E*. Each composite good is also a CES aggregate of individual varieties produced by monopolistically competitive firms. The aggregate production and corresponding price index in each sector is given by:

$$\begin{array}{ccc} U & = & {\left(X_{h}^{\frac{\epsilon -1}{\epsilon }}+X_{l}^{\frac{\epsilon -1}{\epsilon }}\right)}^{\frac{\epsilon }{\epsilon -1}}\\ E & = & w_{h}H+w_{l}L \end{array}$$ (5)

$$\begin{array}{ccc} X_{k} & = & {\left(\int_{0}^{N_{k}}q_{k}{\left(i\right)}^{\frac{\sigma -1}{\sigma }}di\right)}^{\frac{\sigma }{\sigma -1}},\;k\in \left\{h,l\right\} \end{array}$$ (6)

$$\begin{array}{ccc} P_{k} & = & {\left(\int_{0}^{N_{k}}p_{k}{\left(i\right)}^{1-\sigma }di\right)}^{\frac{1}{1-\sigma }},\;k\in \left\{h,l\right\}. \end{array}$$ (7)

We assume that *σ* > *ϵ* > 1 meaning there is a greater substitutability between varieties within each composite good than in final consumption.

On the supply side, firms in each sector behave monopolistically. Firms in sector *h* incur a fixed cost paid in skilled labor, and a unit skilled labor requirement to produce one unit of output. Firms in sector *l* use a different technology. They incur a lower fixed cost paid in skilled labor, and a unit unskilled labor requirement to produce one unit of output. In the appendix we develop the more general case where both sectors use unskilled and skilled labor, but with different intensity. A similar interpretation is to consider a single sector with two types of firms with type-*h* firms intensive in high-skilled workers and type-*l* firms intensive in low-skilled workers. This would be consistent with the results in [41] who highlight the great differences in factor-endowment and technology between firms within sectors, and illustrates the fine line that separates in theory a product from an industry (as pointed out by an anonymous referee). Finally, all firms pay the fixed cost F_*X*_, paid using skilled labor, when selling to the export market. We assume foreign countries are identical, in particular with respect to factor endowment. We also assume, without loss of generality, that there is no variable trade cost. The cost functions of each type of firm are given by:

$$\begin{array}{ccc} TC_{h}\left(q_{h}\right) & = & F_{X}w_{h}+F_{h}w_{h}+q_{h}w_{h}\\ TC_{l}\left(q_{l}\right) & = & F_{X}w_{h}+F_{l}w_{h}+q_{l}w_{l} \end{array}$$

where F_*X*_ is the export fixed cost, and F_*h*_ > F_*l*_. Our objective is to look at the impact of a reduction in the fixed cost on the skilled-unskilled wage gap, and how this changes with the share of type-*l*-firms.

Firms maximize their profit, and we get the standard pricing rule, where firms charge a constant markup over their marginal cost.

$$\begin{array}{ccc} p_{h} & = & \frac{\sigma }{\sigma -1}w_{h} \end{array}$$ (8)

$$\begin{array}{ccc} p_{l} & = & \frac{\sigma }{\sigma -1}w_{l}. \end{array}$$ (9)

Free-entry drives profits to zero and gives us the equilibrium quantities produced by a typical firm in each sector:

$$\begin{array}{ccc} q_{h} & = & \left(F_{X}+F_{h}\right)\left(\sigma -1\right) \end{array}$$ (10)

$$\begin{array}{ccc} q_{l} & = & \left(F_{X}+F_{l}\right)\left(\sigma -1\right)\omega , \end{array}$$ (11)

where *ω* is the wage gap (*ω* = *w*_*h*_/*w*_*l*_). The revenues of a typical *h*-type firms are given by: *R*_*h*_ = *p*_*h*_*q*_*h*_ = (*F*_*X*_ + *F*_*h*_)*σw*_*h*_ and the revenues of a typical *L*-type firms are: *R*_*l*_ = *p*_*l*_*q*_*l*_ = (*F*_*X*_ + *F*_*l*_)*σw*_*h*_. With F_*h*_ greater than F_*l*_, it follows that *R*_*h*_/*R*_*l*_ is greater than one, meaning that *h*-type firms have greater revenues than *l*-type firms.

We can express the aggregate quantities consumed *X*_*h*_ and *X*_*l*_, as well as the corresponding price indices *P*_*h*_ and *P*_*l*_, as a function of the number of *h*-type and *l*-type firms in each sector, *N*_*h*_ and *N*_*l*_:

$$\begin{array}{ccc} X_{k} & = & q_{k}N_{k}^{\frac{\sigma }{1-\sigma }},\;k\in \left\{h,l\right\} \end{array}$$ (12)

$$\begin{array}{ccc} P_{k} & = & p_{k}N_{k}^{\frac{1}{1-\sigma }},\;k\in \left\{h,l\right\}. \end{array}$$ (13)

Relative demand for the two composite goods is given by:

$$\begin{array}{ccc} \frac{X_{h}}{X_{l}} & = & {\left(\frac{P_{h}}{P_{l}}\right)}^{-\epsilon } \end{array}$$ (14)

Substituting Eqs (12) and (13) into Eq (14), and then using Eqs (8)–(11), we can express the wage gap as a function of the ratio of fixed costs and the relative number of firms:

$$\begin{array}{ccc} \frac{q_{h}}{q_{l}}{\left(\frac{N_{h}}{N_{l}}\right)}^{\frac{\sigma }{\sigma -1}} & = & {\left[\frac{p_{h}}{p_{l}}{\left(\frac{N_{h}}{N_{l}}\right)}^{1/(1-\sigma )}\right]}^{-\epsilon }\\ {\left(\frac{F_{X}+F_{l}}{F_{X}+F_{h}}\right)}^{\frac{1}{\epsilon -1}}{\left(\frac{N_{l}}{N_{h}}\right)}^{\frac{\sigma -\epsilon }{\left(\sigma -1)(\epsilon -1\right)}} & = & \omega \end{array}$$ (15)

It is straightforward to see that the derivative of *ω* with respect to F_*X*_ is positive and that the second derivative with respect to F_*X*_ and *N*_*l*_/*N*_*h*_ is also positive. Thus, a lower fixed cost of exporting leads to a reduction in the wage-gap and the effect is stronger the larger is the share of (smaller) *l*-type firms. Note that the number of firms is assumed to be exogenous in this monopolistic competitive model. In the appendix we endogenize the number of firms and solve for a full model with more general assumptions on the production side to show that the wage gap increases with the export fixed costs, and that this effect is larger the larger is the endowment of unskilled to skilled workers.

### 5.2 Empirical evidence

To empirically investigate the role of small firms in cross-border e-commerce, we go back to the harmonized I2D2 and extract information on firm size. We compute, for each country and year the share of workers employed in firms with fewer than 10 employees (information on firm size is unfortunately not available for Colombia, the Dominican Republic, Sri Lanka, and the Philippines). It is on average 45 percent, and it oscillates between 15 percent in Russia and 74 percent in Costa Rica. This suggests that for many countries in our sample the share of employment in small firms is sufficiently large as to have an economically significant impact on the wage gap.

We also compute the share of skilled workers in small and large firms to check whether small firms are less skilled-intensive. Columns (2) and (3) of Table 8 show that on average only 25 percent of workers in small firms (firms with fewer than 10 employees) are considered skilled. In large firms (firms with more than 50 employees) 49 percent of workers are skilled. Comparing each row in columns (2) and (3) of Table 8 we observe in all countries a larger share of skilled workers in large firms.

**Table 8:** Share of employment in small and large firms by country.

Country	Employment share in small firms	Skilled/total in small firms	Skilled/total in large firms
	(1)	(2)	(3)
ARG	29.52	48.61	78.22
BOL	52.42	44.15	71.07
BRA	60.96	39.71	
CHL	26.13	46.98	72.47
CRI	73.96	21.06	
ECU	49.13	8.768	19.06
ETH	59.69	20.13	38.67
GTM	53.92	16.25	34.99
HND	59.79	7.520	48.78
KHM	45.07	12.57	16.44
MEX	41.96	21.75	48.86
NIC	42.33	14.88	50.59
PAN	20.34	28.36	69.74
PER	44.00	53.58	85.46
PRY	43.38	39.10	70.39
RUS	14.61	14.33	19.94
SLV	33.73	4.96	16.10
URY	37.59	17.77	46.43
Average	45.20	25.39	49.20

Source: World Bank’s I2D2 database. Small firms are defined as those with less than 10 employees (excluding self-employment). Large firms as those with more than 50 employees. Column (1) provides the share of workers in small firms in the total labor force. Column (2) provides the share of skilled workers in small firms’ total employment, and column (3) the share of skilled workers in large firms’ total employment. Skilled workers are defined as workers with at least a complete secondary education. There is no information in column (3) for Costa Rica and Brazil because unlike other countries in our sample they do not provide information for workers in firms above 50 employees.

The variation across countries in the share of employment in small firms suggests that if the mechanism at work is the one described above, then the impact of online exports on the wage gap is likely to be heterogeneous across countries. In order to test this heterogeneity and provide evidence for our mechanism, we re-estimate Eq (1) with an interaction between online exports and two dummies indicating whether the country is above or below the median share of employment in small firms in our sample. The median is at 44 percent in our sample. We use a dummy for two reasons. First, the size of the firm is not systematically reported by all individuals in the I2D2 surveys and the resulting information may be quite noisy. In addition, the I2D2 does not provide the exact number of employees, but an interval with a lower and upper bound for firm size. Second, the relationship between the impact of online exports on the wage gap and the share of workers in small firms may not be linear. The solution to these two problems is to use a dummy variable instead of the actual continuous variable. We expect the coefficient on the interaction of online exports with the dummy indicating a share of employment in small firms above the median to be negative and larger than the coefficient on the interaction of online exports with the dummy indicating a share of employment in small firms below the median. Because we are ultimately interested in the interaction between the role of small firms and online exports, we also interact the total exports with the same dummies to ensure that the mechanism we describe is not also present in the case of total exports. If the channel through which exports affect the wage gap works through the ability of small firms to export via online platforms, the interaction terms between total exports and the dummies should not be statistically different from one another.

The estimates are reported in Table 9. The first four columns are estimated using ordinary least squares and the last four using instrumental variables. Columns (1) and (5) correspond to our benchmark specification, whereas columns (2) and (6) replace country fixed effects by country-specific time trends; columns (3) and (7) control for net investment in physical capital. Columns (4) and (8) use only the interactions of online exports and the dummy indicating the above median share of employment in small firms. The interaction between online exports and the dummy is negative and statistically significant in all columns. Note that the dummies indicating a share of employment above and below the median is not time variant, and therefore the impact of the dummies on the skilled-unskilled wage gap is perfectly captured by the country fixed effects. Results are almost identical to the baseline results. Note that the coefficient is very stable across specifications and precisely estimated (p-value below 1%). On the other hand, the interaction with the dummy indicating a share of employment in small firms below the median is not statistically different from zero. Thus, the negative impact of online exports on the wage gap is observed in countries with a larger share of small firms. The impact of total exports on the wage gap remains negative, but becomes statistically insignificant.

**Table 9:** Impact of online exports on the wage gap: The role of small firms.

	OLS	OLS	OLS	OLS	IV	IV	IV	IV
	(1)	(2)	(3)	(4)	(5)	(6)	(7)	(8)
Ln online exports/GDP × above	-0.012***	-0.017***	-0.012***	-0.011***	-0.014***	-0.021*	-0.013***	-0.012***
	(0.002)	(0.003)	(0.003)	(0.002)	(0.005)	(0.012)	(0.004)	(0.001)
Ln online exports/GDP × under	-0.009	-0.012	0.006	-0.008	0.018	0.031	0.028	-0.008
	(0.008)	(0.007)	(0.012)	(0.008)	(0.042)	(0.022)	(0.033)	(0.032)
Ln total exports/GDP × above	-0.048	-0.110**	-0.067		-0.059	-0.113	-0.092*	
	(0.051)	(0.045)	(0.056)		(0.108)	(0.084)	(0.049)	
Ln total exports/GDP × under	-0.012	-0.057*	-0.056		-0.329	-0.398**	-0.089	
	(0.010)	(0.030)	(0.042)		(0.442)	(0.177)	(0.056)	
Ln relative supply	-0.055	0.112	-0.016		-0.057	0.072	0.009	
	(0.033)	(0.074)	(0.032)		(0.110)	(0.090)	(0.033)	
Internet user/Pop.	-0.234	0.437	-0.538		-0.107	1.735	-0.706*	
	(0.248)	(0.347)	(0.434)		(0.977)	(1.212)	(0.368)	
Net investment^a^ (% of GDP)			0.014*				0.016	
			(0.007)				(0.010)	
Observations	105	105	78	105	105	105	78	105
Country FE	Yes	No	Yes	Yes	Yes	No	Yes	Yes
Country time trends	No	Yes	No	No	No	Yes	No	No
Year FE	Yes	Yes	Yes	Yes	Yes	Yes	Yes	Yes

The first four columns are estimated using ordinary least squares, and the last four columns use an instrumental variable estimator. Columns (1),(3) (4),(5),(7) and (8) include country and year fixed effects; Columns (2) and (6) include year fixed effects and country-specific time trends. Standard errors in parenthesis clustered at the country level. Significance levels:

*: 10%,

**: 5%

***: 1%.

^*a*^: Net investment in non-financial assets (% of GDP)

## 6 Concluding remarks

We examine the impact of online exports in reducing the wage gap between skilled and unskilled workers. Our econometric results suggest that as the share of online exports increases the skilled-unskilled wage gap declines. The economic significance of the impact is small as the elasticity of the wage gap with respect to the share of online exports is 0.01, reflecting the fact that online cross-border trade is still a relatively small phenomenon. However, with online cross border trade growing at a pace seven times faster than world trade, its contribution to reductions in wage inequality is bound to increase.

We find that the impact of online exports is stronger in countries where the share of the labor force in small firms is larger. Small firms are more likely to benefit from the reduction in the fixed cost of exporting provided by online markets. They also tend to hire relatively more unskilled workers. Thus, as the share of online exports increases, this leads to a relative increase in the demand faced by small firms, which will in turn increase the relative demand for unskilled workers, and reduces the wage gap. In fact we find that the negative impact is only observed in countries with a large share of the labor force in small firms. In countries where the share of the labor force in small firms is below the median, an increase in online exports has no impact on the wage gap.

These results suggest that reducing barriers to online trade may be desirable for reasons that go beyond economic efficiency, as it may help reduce labor income inequality. Existing barriers to e-commerce include unclear taxation, obscure customs requirements, international payment restrictions, and long customs delays [1]. Solutions to some of these problems already exist, such as the introduction of higher de minimis thresholds under which no customs duties or other taxes (such as VAT) apply, the introduction of streamlined procedures for payment of customs duties and other taxes, such as e-Customs or e-taxation, or a coordination effort by exporting and importing countries to allow for the payment of customs duties online through blockchain technology. The announcement in 2019 by half of WTO members that they will start negotiating e-commerce rules is a first step in helping address these problems.

## Appendix

In this appendix we generalize the model we presented in section 5.1 to allow for more general assumptions on the production side. We also fully close the model and instead of looking at whether the impact of a reduction in the fixed cost of exporting is affected by the number of small and unskilled-intensive *l*-type firms relative to large *h*-type firms (which is an endogenous variable). We explore the results in terms of endowments of unskilled to skilled workers in the economy, which are assumed exogenous and are a determinant of the number of small and large firms.

### Aggregate demand

As in section 5.1 a representative consumer has CES preferences over two imperfectly substitutable composite goods *X*_*h*_ and *X*_*l*_ and maximize their utility with respect to their budget constraint *E*. Each composite good is a CES aggregate of individual varieties produced by monopolistically competitive firms. The aggregate consumption and corresponding price index in each sector are given by:

$$\begin{array}{ccc} U & = & {\left(X_{h}^{\frac{\epsilon -1}{\epsilon }}+X_{l}^{\frac{\epsilon -1}{\epsilon }}\right)}^{\frac{\epsilon }{\epsilon -1}}\\ E & = & w_{h}H+w_{l}L \end{array}$$ (16)

$$\begin{array}{ccc} X_{k} & = & {\left(\int_{0}^{N_{k}}q_{k}{\left(i\right)}^{\frac{\sigma -1}{\sigma }}di\right)}^{\frac{\sigma }{\sigma -1}},\;k\in \left\{h,l\right\} \end{array}$$ (17)

$$\begin{array}{ccc} P_{k} & = & {\left(\int_{0}^{N_{k}}p_{k}{\left(i\right)}^{1-\sigma }di\right)}^{\frac{1}{1-\sigma }},\;k\in \left\{h,l\right\}. \end{array}$$ (18)

We assume that *σ* > *ϵ* > 1 meaning there is a greater substitutability between varieties within each composite good than in final consumption.

### Firm production

Firms behave monopolistically and incur a production and export fixed cost to be paid in skilled labor, and a variable using a mix of skilled and unskilled labor. Type-*h* firms incur a production fixed cost *F*_*h*_ and require *α*_*h*_ skilled-labor and 1 − *α*_*h*_ unskilled labor to produce one unit of output. Type-*l* firms incur a lower production fixed cost F_*l*_ and require *α*_*l*_ skilled labor and 1 − *α*_*l*_ unskilled workers. We assume that firms in sector *h* are more skill-intensive in production than firms in sector *l*, i.e. *α*_*h*_ > *α*_*l*_. All firms incur the export fixed cost F_*X*_. The cost functions of a typical firm in each sector are given by:

$$\begin{array}{ccc} TC_{h}\left(q_{h}\right) & = & F_{X}w_{h}+F_{h}w_{h}+q_{h}w_{h}^{\alpha_{h}}w_{l}^{1-\alpha_{h}}\\ TC_{l}\left(q_{l}\right) & = & F_{X}w_{h}+F_{l}w_{h}+q_{l}w_{h}^{\alpha_{l}}w_{l}^{1-\alpha_{l}} \end{array}$$

Firms maximize their profit, and we get the standard pricing rule, where firms charge a constant markup over their marginal cost.

$$\begin{array}{cc} p_{h} & =\frac{\sigma }{\sigma -1}w_{h}^{\alpha_{k}}w_{l}^{1-\alpha_{k}} \end{array}$$ (19)

$$\begin{array}{cc} p_{l} & =\frac{\sigma }{\sigma -1}w_{h}^{\alpha_{l}}w_{l}^{1-\alpha_{l}} \end{array}$$ (20)

Free-entry in each sector drives profits to zero and gives us the equilibrium quantities produced by a typical *h*-type and *l*-type firm:

$$\begin{array}{c} q_{h}=\left(F_{X}+F_{h}\right)\left(\sigma -1\right)\omega^{1-\alpha_{h}} \end{array}$$ (21)

$$\begin{array}{c} q_{l}=\left(F_{X}+F_{l}\right)\left(\sigma -1\right)\omega^{1-\alpha_{l}}, \end{array}$$ (22)

where *ω* is the wage gap between skilled and unskilled worker (i.e. the wage gap *ω* = *w*_*h*_/*w*_*l*_). Note that as long as F_*h*_ is greater than F_*l*_, the typical *h*-type firms has greater revenues than its *l*-type counterpart.

### Labor market clearing conditions

To close the model we need to determine the number of firms active in each sector, which we get from the labor market clearing conditions. Skilled labor is used to pay the fixed cost of firms in both sector, and used with intensity *α*_*h*_ in sector *h*, and with intensity *α*_*l*_ in the *l* sector. Unskilled labor is only used in the production of each firm with the complementary intensities 1 − *α*_*h*_ in sector *h* and 1 − *α*_*l*_ in sector *l*. Using the total cost functions and Shephard’s lemma, we obtain the following demand for skilled and unskilled labor:

$$\begin{array}{ccc} H & = & N_{h}\left(F_{X}+F_{h}\right)\left(1+\alpha_{h}\left(\sigma -1\right)\right)+N_{l}\left(F_{X}+F_{l}\right)\left(1+\alpha_{l}\left(\sigma -1\right)\right) \end{array}$$ (23)

$$\begin{array}{ccc} L & = & N_{h}\left(F_{X}+F_{h}\right)\omega \left(\sigma -1\right)\left(1-\alpha_{h}\right)+N_{l}\left(F_{X}+F_{l}\right)\omega \left(\sigma -1\right)\left(1-\alpha_{l}\right) \end{array}$$ (24)

The equilibrium is given by the 11 Eqs (17)–(24) and the 11 unknowns *E*, *X*_*h*_, *X*_*l*_, *P*_*h*_, *P*_*l*_, *p*_*h*_, *p*_*l*_, *q*_*h*_, *q*_*l*_, *N*_*h*_, *N*_*l*_.

### Solving for the wage gap

Our aim is to express the wage gap (*ω*) as a function of the relative factor endowment ($\frac{H}{L}$) and the fixed costs *F*_*h*_, *F*_*l*_, *F*_*X*_. The labor market clearing conditions allow us to pin down the number of *h*-type and *l*-type firms. Using Eqs (23) and (24) we get:

$$\begin{array}{ccc} N_{h} & = & \frac{H\omega \left(\sigma -1\right)\left(1-\alpha_{l}\right)-L\left(1+\alpha_{l}\left(\sigma -1\right)\right)}{\left(F_{X}+F_{h}\right)\omega \sigma \left(\sigma -1\right)\left(\alpha_{h}-\alpha_{l}\right)} \end{array}$$ (25)

$$\begin{array}{ccc} N_{l} & = & \frac{H\omega \left(\sigma -1\right)\left(\alpha_{h}-1\right)+L\left(1+\alpha_{h}\left(\sigma -1\right)\right)}{\left(F_{X}+F_{l}\right)\omega \sigma \left(\sigma -1\right)\left(\alpha_{h}-\alpha_{l}\right)} \end{array}$$ (26)

For convenience, let us define the ratio of the number of firms as a function of the wage gap *ω* and relative factor endowment $\frac{H}{L}$. We define the function *g* such that: $\frac{N_{h}}{N_{l}}=\frac{F_{X}+F_{l}}{F_{X}+F_{h}}g\left(\omega ,\frac{H}{L}\right)$ where $g\left(\omega ,\frac{H}{L}\right)=\frac{\frac{H}{L}\omega \left(\sigma -1\right)\left(1-\alpha_{l}\right)-\left(1+\alpha_{l}\left(\sigma -1\right)\right)}{\frac{H}{L}\omega \left(\sigma -1\right)\left(\alpha_{h}-1\right)+\left(1+\alpha_{h}\left(\sigma -1\right)\right)}$. Note that the function *g* is strictly positive and different from zero as it is defined as the ratio of the number of firms. CES preferences over the varieties in each sector ensures that there is at least one firm producing in each sector. Using CES preferences in the consumption of the aggregate goods gives the usual relationship between relative aggregate consumption and relative price indices:

$$\begin{array}{ccc} \frac{X_{h}}{X_{l}} & = & {\left(\frac{P_{h}}{P_{l}}\right)}^{-\epsilon }. \end{array}$$ (27)

Within each sector, the CES structure of consumption of varieties allows us to express the aggregate quantities consumed *X*_*h*_ and *X*_*l*_ as well as the corresponding price indices as a function of the number of firms in each sector:

$$\begin{array}{ccc} X_{k} & = & q_{k}N_{k}^{\frac{\sigma }{1-\sigma }},\;k\in \left\{h,l\right\} \end{array}$$ (28)

$$\begin{array}{ccc} P_{k} & = & p_{k}N_{k}^{\frac{1}{1-\sigma }},\;k\in \left\{h,l\right\}. \end{array}$$ (29)

Substituting for the equilibrium prices and quantities into Eqs (28) and (29) and plugging into Eq (27), we can express the wage gap as a function of relative factor endowment, a function of the export fixed cost $\frac{F_{X}+F_{l}}{F_{X}+F_{h}}$, and the relative number of firms:

$$\begin{array}{ccc} \frac{X_{h}}{X_{l}} & = & {\left(\frac{P_{h}}{P_{l}}\right)}^{-\epsilon }\\ \frac{q_{h}}{q_{l}}{\left(\frac{N_{h}}{N_{l}}\right)}^{\frac{\sigma }{\sigma -1}} & = & {\left[\frac{p_{h}}{p_{l}}{\left(\frac{N_{h}}{N_{l}}\right)}^{1/(1-\sigma )}\right]}^{-\epsilon }\\ g{\left(\omega ,\frac{H}{L}\right)}^{\frac{\sigma -\epsilon }{\epsilon -1}}\omega^{\left(\alpha_{h}-\alpha_{l}\right)\left(\sigma -1\right)} & = & \frac{F_{X}+F_{l}}{F_{X}+F_{h}} \end{array}$$ (30)

This last expression links the wage gap *ω* to factor endowment $\frac{H}{L}$ and to the difference in fixed cost requirement between type-*h* and type-*l* firms and the export fixed cost *F*_*X*_. We are primarily interested in how the fixed cost of exporting affects the wage gap. To ease the reading, we call $h\left(F_{X}\right)=\frac{F_{X}+F_{l}}{F_{X}+F_{h}}$. Note that *h*′(*F*_*X*_) is positive since *F*_*h*_ − *F*_*l*_ is positive.

We now use the implicit function theorem and totally differentiate this expression. Using hat-algebra (i.e. $\hat{x}=\frac{dx}{x}$) we obtain:

$$\begin{array}{ccc} \hat{\omega } & = & \frac{1}{\left(\sigma -1\right)\left(\alpha_{h}-\alpha_{l}\right)+\frac{\sigma -\epsilon }{\epsilon -1}\varepsilon_{g,\omega }}\hat{h}\left(F_{X}\right)-\frac{\frac{\sigma -\epsilon }{\epsilon -1}\varepsilon_{g,H/L}}{\left(\sigma -1\right)+\frac{\sigma -\epsilon }{\epsilon -1}\varepsilon_{g,\omega }}\hat{\frac{H}{L}}, \end{array}$$ (31)

where *ε*_*g*,*ω*_ and *ε*_*g*,*H*/*L*_ are the elasticities of the *g* function with respect to *ω* and $\frac{H}{L}$ respectively. It is straightforward to check that these two elasticities are identical: *ε*_*g*,*ω*_ = *ε*_*g*,*H*/*L*_ are equal to:

$$\begin{array}{ccc} \varepsilon_{g,\omega }=\varepsilon_{g,H/L} & = & \frac{\omega \frac{H}{L}\sigma \left(\sigma -1\right)\left(\alpha_{h}-\alpha_{l}\right)}{{\left[\frac{H}{L}\omega \left(\sigma -1\right)\left(\alpha_{h}-1\right)+\left(1+\alpha_{h}\left(\sigma -1\right)\right)\right]}^{2}}\frac{1}{g(\omega ,H/L)}\\ & = & \frac{\frac{H}{L}\omega \sigma \left(\sigma -1\right)\left(\alpha_{h}-\alpha_{l}\right)}{\left(\frac{H}{L}\omega \left(\sigma -1\right)\left(\alpha_{h}-1\right)+1+\alpha_{h}\left(\sigma -1\right))(\frac{H}{L}\omega \left(\sigma -1\right)\left(\alpha_{l}-1\right)-1-\alpha_{l}\left(\sigma -1\right)\right)} \end{array}$$

These two elasticities are strictly positive since we imposed *α*_*h*_ > *α*_*l*_ > 0 and *σ* > 1. It follows that in Eq (31), the terms in front of $\hat{h}\left(F_{X}\right)$ and $\hat{\frac{H}{L}}$ are positive. Therefore, a reduction in the fixed cost of exporting reduces the wage gap between skilled and unskilled workers. Similarly, an increase in the relative supply of skilled workers also reduces the wage gap. Finally, we want to investigate whether the marginal effect of a reduction in the fixed cost of varies with factor endowment. We simply take the second order derivative of the wage gap with respect to *h*(*F*_*X*_) and to the relative factor endowment:

$$\begin{array}{ccc} \frac{\partial \omega }{\partial h(F_{X})} & = & \frac{\omega^{1-\left(\alpha_{h}-\alpha_{l}\right)\left(\sigma -1\right)}g{\left(\omega ,H/L\right)}^{-\frac{\sigma -\epsilon }{\epsilon -1}}}{\left(\sigma -1\right)\left(\alpha_{h}-\alpha_{l}\right)+\frac{\sigma -\epsilon }{\epsilon -1}\varepsilon_{g,\omega }}\\ \frac{\partial^{2}\omega }{\partial h(F_{X})\partial H/L} & = & -\frac{(\frac{\sigma -\epsilon }{\epsilon -1})\omega^{1-\left(\alpha_{h}-\alpha_{l}\right)\left(\sigma -1\right)}g{\left(\omega ,H/L\right)}^{-\frac{\sigma -\epsilon }{\epsilon -1}}}{{\left[\left(\sigma -1\right)\left(\alpha_{h}-\alpha_{l}\right)+\frac{\sigma -\epsilon }{\epsilon -1}\varepsilon_{g,\omega }\right]}^{2}}[\frac{L}{H}\varepsilon_{g,H/L}(\left(\sigma -1\right)\left(\alpha_{h}-\alpha_{l}\right)\\ & & +\frac{\sigma -\epsilon }{\epsilon -1}\varepsilon_{g,\omega })+\frac{\partial \varepsilon_{g,\omega }}{\partial H/L}]. \end{array}$$

A sufficient condition for this equation to be negative is for the partial derivative $\frac{\partial \varepsilon_{g,\omega }}{\partial H/L}$ to be positive since the other terms between brackets are positive. Deriving the elasticity gives us the following expression, which is positive given the restriction imposed on factor-intensity (*α*_*h*_ > *α*_*l*_ > 0).

$$\begin{array}{ccc} \frac{\partial \varepsilon_{g,\omega }}{\partial H/L} & = & \frac{\left(\sigma -1\right)\left(\alpha_{h}-\alpha_{l}\right)\left(1-\alpha_{h}\right)\omega }{\alpha_{l}{\left[\frac{H}{L}\omega \left(\sigma -1\right)\left(\alpha_{h}-1\right)+1+\alpha_{h}\left(\sigma -1\right)\right]}^{2}}. \end{array}$$
